# Rare *TBK1* variants in patients with frontotemporal dementia and amyotrophic lateral sclerosis in a Chinese cohort

**DOI:** 10.1186/s40035-018-0136-6

**Published:** 2018-12-04

**Authors:** Bin Jiao, Qiying Sun, Zhenhua Yuan, Junling Wang, Lin Zhou, Xinxiang Yan, Beisha Tang, Lu Shen

**Affiliations:** 1 Department of Neurology, Xiangya Hospital, Central South University, 87 Xiangya Rd, Changsha, 410008 China; 2 National Clinical Research Center for Geriatric Disorders, Central South University, Changsha, China; 3 Key Laboratory of Hunan Province in Neurodegenerative Disorders, Central South University, Changsha, China; 4 Department of Geriatrics Neurology, Xiangya Hospital, Central South University, Changsha, China; 5 Parkinson’s Disease Center of Beijing Institute for Brain Disorders, Beijing, China; 6 Collaborative Innovation Center for Brain Science, Shanghai, China; 7 Collaborative Innovation Center for Genetics and Development, Shanghai, China; 8 Key Laboratory of Organ Injury, Aging and Regenerative Medicine of Hunan Province, Changsha, China

**Keywords:** Amyotrophic lateral sclerosis, Frontotemporal dementia, *TBK1 gene*

## Abstract

**Background:**

The TANK-Binding Kinase 1 (*TBK1*) gene has recently been identified as the third or fourth most frequent cause of frontotemporal dementia (FTD) and amyotrophic lateral sclerosis (ALS). The aim of this study was to assess the genetic contribution of *TBK1* in a Chinese cohort.

**Methods:**

A total of 270 cases with ALS, FTD, or their combination were recruited into this study. All the coding exons of *TBK1* and intron-exon boundaries were sequenced using Sanger sequencing. The frequency of *TBK1* variants and the correlation with clinical phenotypes were analyzed.

**Results:**

A novel mutation (c.1959_1960insGT, p.E653fs) was identified in a sporadic case with semantic dementia, secondarily developing ALS. Another novel variant (c.2063_2064delTT, p.L688Rfs*14) was found in an ALS-FTD family. Totally, the *TBK1* variants could only account for 0.7% of cases.

**Conclusions:**

This study enlarges the genetic and phenotypic spectrum of *TBK1* mutation in a Chinese cohort. Our data indicates that *TBK1* mutation is not a common cause for ALS and FTD in Chinese patients.

**Electronic supplementary material:**

The online version of this article (10.1186/s40035-018-0136-6) contains supplementary material, which is available to authorized users.

## Background

Amyotrophic lateral sclerosis (ALS) is a multisystem degenerative condition clinically characterized by the predominant loss of motor neurons and progressive weakness of voluntarily innervated muscles, including muscles of the respiratory apparatus. Patients eventually die of respiratory failure within 3–5 years [1]. Frontotemporal dementia (FTD) is a focal clinical syndrome characterized by behavior changes and language impairment, associated with circumscribed degeneration of the prefrontal and anterior temporal cortex. Onset is typically in the middle years of life and survival is approximately 8 years [2]. Traditionally, ALS and FTD have been considered as two different neurodegenerative diseases; however, recent studies have indicated that both shared common clinical features, pathologic spectrum, and causative genes [3, 4]. Approximately 10% of patients with FTD presented clinical evidence of ALS at some stage in the disease course; conversely, above 10% to 15% of patients with ALS showed behavioral changes and/or language dysfunctions that met the diagnosis of FTD [5, 6]. Additionally, accumulation of TAR DNA-binding protein 43 (TDP-43) positive inclusions in the brain regions and motor neurons have been detected in both patients with FTD and ALS, which were considered as common neuropathology of both disorders [7]. In addition, the identification of *TARDBP* as the first shared ALS/FTD gene was followed by a wave of discovered causative genes, such as chromosome 9 open reading frame 72 (*C9orf72*), valosin-containing protein (*VCP*), which could cause both ALS and FTD [8–10]. In summary, ALS and FTD have been increasingly regarded as part of a disease spectrum.

Recently, the TANK-binding kinase 1 (*TBK1*) gene was identified to be highly associated with ALS and FTD [11]. So far, at least 100 variants in the *TBK1* gene, including loss of function variants, in-frame deletions of single amino acids, and missense variants, have been reported in ALS, FTD, or ALS-FTD patients, thus making *TBK1* the third or fourth most frequent genetic cause of ALS and FTD [12]. TBK1 protein is a multifunctional kinase, which is known to bind to and phosphorylate a series of proteins involved in innate immunity and autophagy, including optineurin (OPTN) and p62, both of which have been implicated in the process of ALS [13, 14].

In this study, we screened a cohort of 270 cases with ALS, FTD and ALS-FTD from the Chinese Han population, and then determined the *TBK1* variant frequencies and the correlation with clinical phenotypes.

## Methods

### Subjects

A total of 270 cases were recruited into this study, including 180 cases with ALS (age of onset: 47.5 ± 13.0 years; male: 70.0%; family history: 9.4%), 88 cases with FTD (age of onset: 56.1 ± 10.1 years; male: 42.1%; family history: 12.5%), a sporadic case with FTD-ALS (age at onset: 61 years; female), and an ALS-FTD family (age at onset of proband: 45 years; male). Mutations in the most common causative ALS genes (*SOD1*, *TARDBP*, *FUS*, *C9orf72*) and FTD genes (*MAPT*, *GRN*, *C9orf72*) were excluded by Sanger sequencing or repeat-primed polymerase chain reaction (PCR, for *C9orf72*). All cases were enrolled from outpatients and inpatients of the department of Neurology, Xiangya Hospital, Central South University. All ALS cases met the EI Escorial criteria for clinical ALS diagnosis [15], and all FTD cases were diagnosed according to the consensus criteria for FTD [16]. The 300 healthy individuals (age mean: 68.8 ± 9.0 years; male: 63.3%) were collected from Xiangya Hospital Wellness Center, and their Mini-Mental State Examination (MMSE) scores were equal or above 28 points. The study was approved by the Ethics Committee of Xiangya Hospital of the Central South University in China (equivalent to an Institutional Review Board). Written informed consent was obtained from each participant.

### Gene screening and genotyping

Genomic DNA was extracted from peripheral blood leukocytes using standard methods. The quality and quantity of DNA were assessed with a fluorometer. All DNA samples were normalized to 50 ng/ul. All subjects were screened for all exons of *TBK1* (NM_013254) using Sanger sequencing. All primers designed by Primer 5 software, were applied to amplify coding regions and flanking non-coding regions of *TBK1* using PCR. The primers and PCR reaction conditions are listed in Additional file 1: Table S1. PCR products were sequenced using identical forward and reverse primers with BigDye terminator v3.1 sequencing chemistry on an ABI 3730xl DNA analyzer (Applied Biosystems). The DNA sequences were then analyzed by Sequencher software, version 4.2.

## Results

**Fig. 1:**
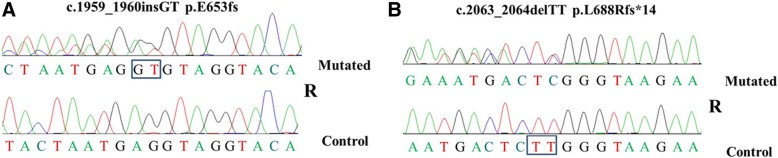
Sanger sequencing showed two nover *TBK1* novel mutations. **a** mutation c.1959_1960insGT, p.E653fs in a sporadic case with FTD-ALS; **b** mutation c.2063_2064delTT, p.L688Rfs*14 in an ALS-FTD family

In this study, we respectively identified two novel mutations c.1959_1960insGT, p.E653fs and c.2063_2064delTT, p.L688Rfs*14 in a sporadic case with FTD-ALS and in an ALS-FTD family (Fig.1a and b). These two variants were not detected in 300 healthy controls, and were also absent in the following database: the HGMD database (*http://www.hgmd.cf.ac.uk/ac*), the Exome Aggregation Consortium (*http://exac.broadinstitute.org*), NHLBI Exome Sequencing Project ((*http://evs.gs.washington.edu/EVS*), the dpSNP database (*http://www.ncbi.nlm.nih.gov/snp*), and the 1000 Genomes Project (result in different data set: Asian, 2012 Apr; CHB (Northern Chinese); JPT (Japanese); CHS (Southern Han Chinese). These two mutations were also predicted to have disease-causing effects using online prediction software (http://sift.jcvi.org/).

**Fig. 2:**
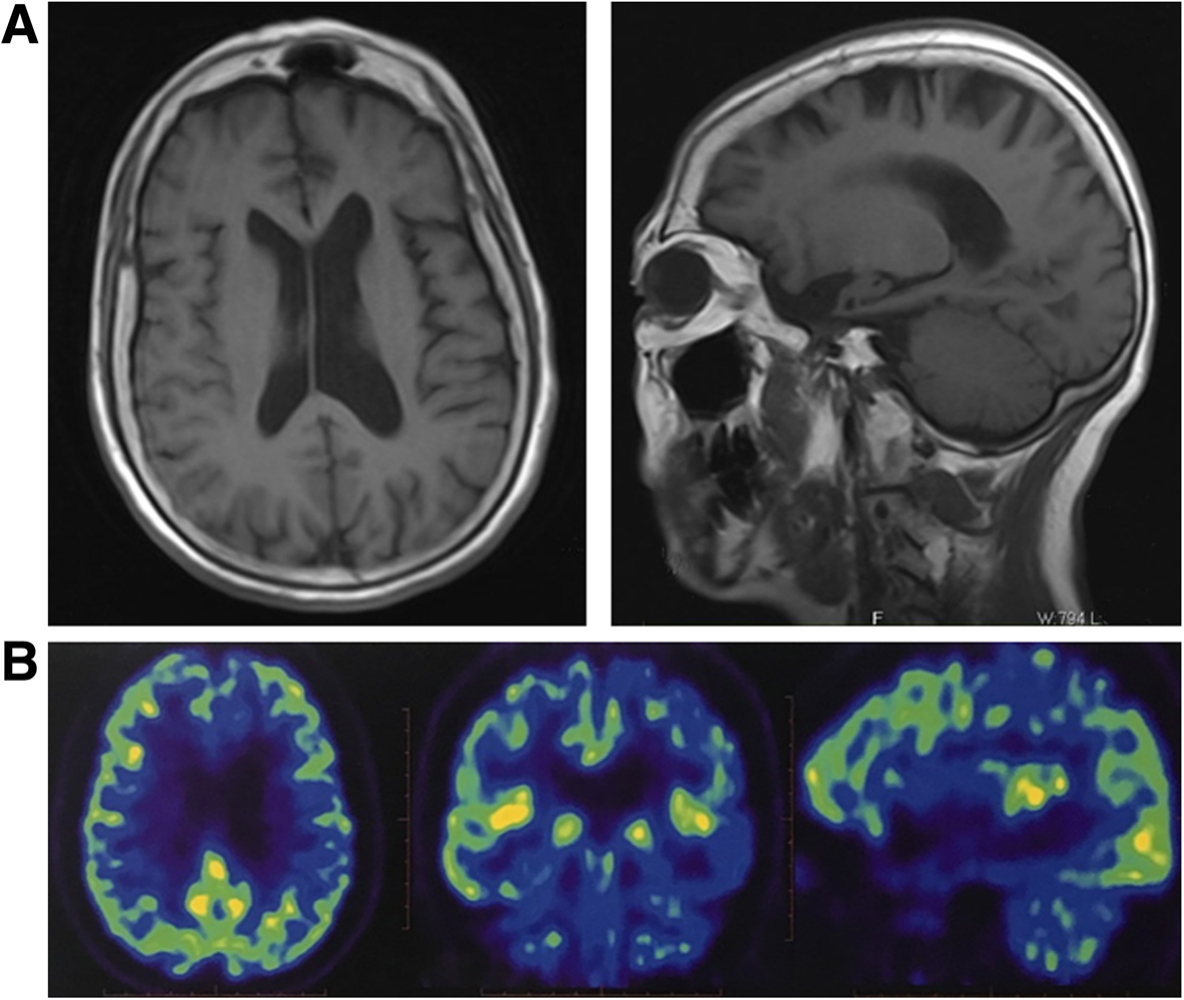
The brain neuroimaging of SD-ALS patient carrying p.E653fs mutation. **a** MRI showed left temporal lobe atrophy;**b** FDG-PET indicated that glucose metabolism decreased in the left temporal lobe and hippocampus

The p.E653fs mutation carrier was a 63-year-old female with a 2-year history of disease. At 61 years of age, the patient presented initial symptoms such as difficulties in finding words and reduced word comprehension. The patient’s husband could not understand her speech due to the use of meaningless words. She also could not say the names of items, such as “key”, “watch”, etc., which were familiar in her daily life. One year later, she experienced progressive weakness in the right limbs, and the symptoms of dysphagia and dysarthria occurred in the last 2 months. Neurologic examination revealed hyperreflexia in the right side; muscle testing showed a score of 4/5 points in proximity and extremity of right side muscles. Hoffmann and Babinski signs were both positive in the right side. The electromyogram (EMG) showed spontaneous denervation activity in the affected limbs. With regard to cognitive assessment, at the beginning, her Mini-Mental State Examination (MMSE) score was 21/30 points, and her Montreal Cognitive Assessment (MoCA) score was 11/30 points; however, 2 years later, she could not finish the above cognitive assessment. Magnetic resonance imaging (MRI) of the brain reported brain atrophy, especially in left temporal lobe (Fig. 2a). The fluorodeoxyglucose positron emission tomography (FDG-PET) indicated that glucose metabolism decreased in left temporal lobe and hippocampus (Fig. 2b). In summary, this patient was clinically diagnosed with semantic dementia (SD), a subtype of FTD, secondarily developing ALS. Unfortunately, it was not possible to verify segregation of the variant with the disease in the family, because the patient had no information about her birth family.

**Fig. 3:**
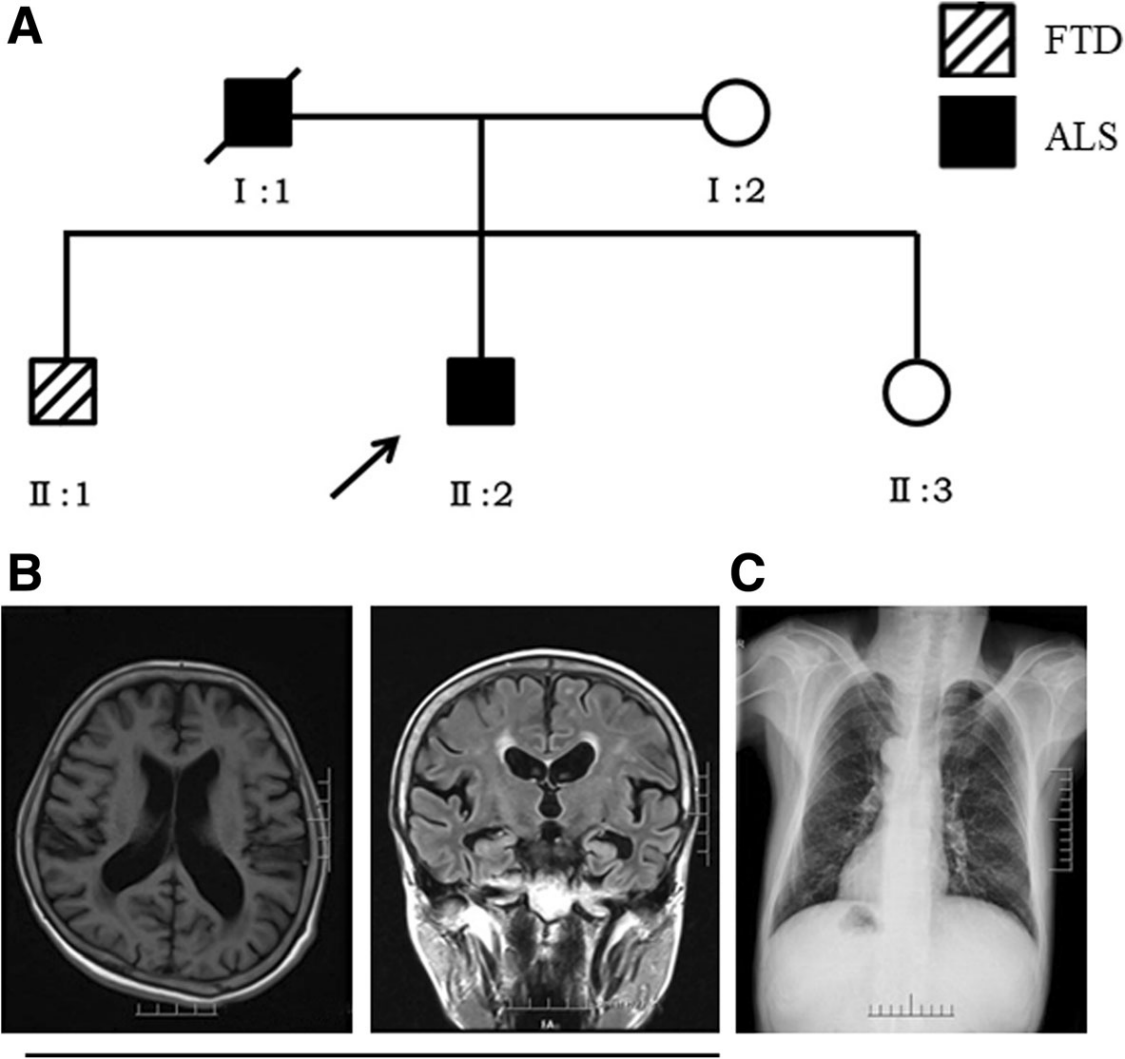
(**a**) The pedigree of the ALS-FTD family with L688Rfs*14 variant, the proband is indicated with an arrow; (**b**) brain MRI of the II:1 case reported frontotemporal lobe and hippocampus atrophy; (**c**) chest X-ray of the II:1 case reported that he had mirror image dextrocardias with situs inversus viscera

The L688Rfs*14 variant was identified in an ALS-FTD family (Fig. 3a). The proband was a 45-year-old male (II: 2), who initially presented left upper limb weakness and atrophy, which extended bilaterally to the neck, shoulder and right upper limb within 4 months. At the time of diagnosis, 6 months after the clinical onset, he had both upper and lower motor signs, slight dysarthria and dysphagia. The EMG showed extensive neurogenic damage, involving brainstem and cervicothoracic and lumbosacral segments. The general cognition assessment was normal (MMSE: 30, MoCA: 29). His father (I: 1) had similar symptoms to those of the proband, and died when he was 44 years old. His 50-year-old brother (II: 1), who also carried that L688Rfs*14 mutation, had been bright and sociable, however, at 36 years of age, he presented progressively bizarre behaviors including withdrawal from social engagements and dishevelment in public. Brain MRI indicated frontotemporal lobe and hippocampus atrophy (Fig. 3b). We also found that he had the mirror image dextrocardias with situs inversus viscera (Fig. 3c). Their mother (I: 2) without similar symptoms did not carry this mutation.

## Discussion

In this study, we screened a Chinese cohort of 270 cases with ALS, FTD, FTD-ALS (or ALS-FTD) for *TBK1* mutations and identified two novel frame-shift *TBK1* mutations (p.E653fs and p.L688Rfs*14) in a sporadic case with SD-ALS and an ALS-FTD family respectively, accounting for 0.7% of total cases, which were consistent with the recent Asian population studies from Taiwan (0.5%; 1/207 ALS) and China (0.7%; 2/294 ALS) [17, 18], although they were significantly lower than European cohorts, such as Italian (3.2%; 5/154 ALS) and Belgian (1.7% 11/629 ALS, FTD, FTD-ALS) [19, 20]. Our results further indicated that the *TBK1* gene was not a common gene in the Chinese population.

The *TBK1* gene contains four domains, N-terminal kinase domain (KD), ubiquitin-like domain (ULD), a-helical scaffold dimerization domain (SDD), and C-terminal domain (CTD) [21]. The two mutations p.E653fs and p.L688Rfs*14 identified in this study were both located in the CTD region, which were critical in TBK1 protein function. A previous study confirmed that the OPTN binding region (amino acids: 601–729) was located in CTD, therefore, we speculate that these two variants have probably lost their ability to interact with OPTN, thus leading to ALS or FTD.

All *TBK1* variants were summarized as three types: loss of function (LoF) variants, in-frame deletions of single amino acids, and missense variants. LoF variants resulting in a 50% reduction of TBK1, could explain 0.4–3.4% of ALS, 0.2–1.3% of FTD and 3.3–4.5% of FTD–ALS [22]. The LoF variants involved premature termination codons (PTCs), insertions, deletions, or splice site mutations resulting in a frame-shift or an in-frame deletion of multiple amino acids [12]. Therefore, the two mutations p.E653fs and p.L688Rfs*14 identified in this study both belong to LoF variants, further implicating their pathogenicity. Further studies should focus on whether these two mutations decrease the *TBK1* mRNA and protein levels.

With regard to the *TBK1* genotype-phenotype relationship, more than half of patients with LoF variants were clinically diagnosed with pure motor neuron disease (MND), which mainly comprised ALS, rare cases with progressive bulbar palsy. Approximately one quarter of *TBK1* mutation carriers were diagnosed with pure FTD, a few with unspecified dementia and above one fifth with a combination of ALS and FTD or unspecified dementia [23]. Interestingly, the two mutations detected in this study were both from cases with FTD combined with ALS. Through reviewing previous studies, we found a total of 54 index patients and 33 affected relatives with an LoF mutation in *TBK1* for whom clinical information was available. Among them, seven variants, including Val479GlufsX4, Ala417X, 690_713del, Arg440X, Arg444X, Thr79del, Thr462LysfsX3 and Glu643del, were identified in ALS-FTD or FTD-ALS cases [11, 12, 18, 23, 24]. Therefore, this study enlarged the *TBK1* genotype. Interestingly, four patients with TBK1 FTD–ALS from French–Portuguese cohort predominantly had language symptoms [25], secondarily developing ALS, which was similar to the p.E653fs mutation carriers in this study, suggesting that we should screen the *TBK1* gene in patients who were clinically diagnosed as SD-ALS.

## Conclusions

In summary, we found two novel *TBK1* LoF variants in a Chinese cohort, which enlarged the gene mutation and clinic phenotype spectrum. To the best of our knowledge, this is the first report of FTD combined with ALS carrying the *TBK1* mutation in the Chinese population, indicating that we should not neglect screening for the *TBK1* gene when encountering the FTD-ALS or ALS-FTD cases.

## Additional file

Additional file 1: **Table S1.** PCR conditions and primer sequences of *TBK1* gene. (DOCX 17 kb)

## Abbreviations

ALS: Amyotrophic lateral sclerosis
*C9orf72*: chromosome 9 open reading frame 72
CTD: C-terminal domain
FTD: Frontotemporal dementia
*FUS*: fused in sarcoma
*GRN*: granulin precursor
KD: kinase domain
LOF: loss of function
*MAPT*: microtubule associated protein tau
MMSE: Mini-Mental State Examination
MND: motor neuron disease
MoCA: Montreal Cognitive Assessment
PCR: polymerase chain reaction
PTCs: premature termination codons
SDD: scaffold dimerization domain
*SOD1*: superoxide dismutase 1
*TBK1*: TANK-binding kinase 1
TDP-43: TAR DNA-binding protein 43
ULD: ubiquitin-like domain
*VCP*: valosin-containing protein
