# Supplementary material for: Rare TBK1 variants in patients with frontotemporal dementia and amyotrophic lateral sclerosis in a Chinese cohort
Source: Transl Neurodegener. 2018 Dec 4;7:31. doi: 10.1186/s40035-018-0136-6 (PMC6278101; doi:10.1186/s40035-018-0136-6)
Supplement: Supplementary file 1 — Table S1. PCR conditions and primer sequences of TBK1 gene. (DOCX 17 kb) [file 40035_2018_136_MOESM1_ESM.docx]

**Table S1:** **PCR conditions and primer sequences of *TBK1***

| ***TBK1* exon** | **Primer sequence** | **Size in bp** | **T_m_ in ℃** |
| --- | --- | --- | --- |
| Exon2 | F: AGACAAGGTAACACCAAAGA  R: CAAAATCACCAAGTCACACA | 557 | Touch down |
| Exon3 | F:TCCCTGTGCCTAAAAGATGC  R: CCGCCCCCTTTTATTTCTT | 484 | Touch down |
| Exon4 | F: AGTCTCAATCTCTTGACCTC  R: AGCCACTTGTGTATCTTCTT | 747 | Touch down |
| Exon5 | F:TTGAGACATGCACACATACACG  R:TGGCTGACCAATTACCCAAT | 343 | Touch down |
| Exon6 | F: AAAACTGAGGATCAAAGGAG  R: CAATAAGATGAACCAAGACA | 423 | Touch down |
| Exon7 | F: AACTTATTTTGTGTTTGCTC  R: AATGTATGCCCTGTGTCCTA | 663 | Touch down |
| Exon8 | F: CATACACCTATAATCCCACC  R: ATCAATCCTTTTACTCCAAA | 713 | Touch down |
| Exon9 | F:CCTCTCACTTTATCCCCAGTT  R: GGCTGTATTTTTCTAATGGCTAGT | 300 | Touch down |
| Exon10-11 | F:ATTGGACTCATTGTGTTGGTGT  R: GGGGCAAGGTCTACATGAAA | 850 | Touch down |
| Exon12 | F:CATCAGGATCACAGAAATGCTT R:GCCATATTGTGCCAAGGATAA | 365 | Touch down |
| Exon13 | F:TCTCCATCTCCTAACTTCGT  R: ACTTGCTTTAACTGCCACAG | 793 | Touch down |
| Exon14-15 | F:CATTTCTGGCTTTTGGCAAT  R:CCTTTTTCCTGACAGAATCACA | 481 | Touch down |
| Exon16 | F:TTACAGACCAAATAAAAGCA  R: GCAGCAAGAAGATAAAGAGA | 593 | Touch down |
| Exon17-18 | F:CCTTCTCTTTATCTTCTTGCTGCT  R:GGGTGGAACTGAATGTAGGC | 598 | Touch down |
| Exon19-20 | F:AAAAGCTGTAACACTTGATGTCAGA R:TGACCCTTTACCACTGCTGA | 595 | Touch down |
| Exon21 | F: ACCATGCTGTTCCAGTAAGAAA  R: CATCTTCACAGCAGCCAAAA | 394 | Touch down |

PCR reaction conditions of all exons: 95℃ 5min-(94℃ 30s- annealing at 62.0℃ and dropping 0.5℃for each cycle 1min -72℃ 1min) *15cycles-（94℃ 30s -56℃ 1min- 72℃ 1min）*28cycles-72℃ 10min.
